# CT texture analysis in patients with locally advanced rectal cancer treated with neoadjuvant chemoradiotherapy: A potential imaging biomarker for treatment response and prognosis

**DOI:** 10.1371/journal.pone.0182883

**Published:** 2017-08-10

**Authors:** Choong Guen Chee, Young Hoon Kim, Kyoung Ho Lee, Yoon Jin Lee, Ji Hoon Park, Hye Seung Lee, Soyeon Ahn, Bohyoung Kim

**Affiliations:** 1 Department of Radiology, Seoul National University Bundang Hospital, Seoul National University College of Medicine, Institute of Radiation Medicine, Seoul National University Medical Research Center, Seongnamsi, Korea; 2 Department of Pathology, Seoul National University Bundang Hospital, Seoul National University College of Medicine, Seongnam-si, Republic of Korea; 3 Medical Research Collaborating Center, Seoul National University Bundang Hospital, Seongnam-si, Republic of Korea; 4 Division of Biomedical Engineering, Hankuk University of Foreign Studies, Yongin-si, Republic of Korea; University of Nebraska Medical Center, UNITED STATES

## Abstract

**Purpose:**

To evaluate the association of computed tomography (CT) texture features of locally advanced rectal cancer with neoadjuvant chemoradiotherapy (CRT) response and disease-free survival (DFS).

**Methods and findings:**

The institutional review board approved this retrospective study. 95 patients who received neoadjuvant CRT, followed by surgery, for locally advanced rectal cancer were included. Texture features (entropy, uniformity, kurtosis, skewness, and standard deviation) were assessed in pretreatment CT images and obtained without filtration and with Laplacian of Gaussian spatial filter of various filter values (1.0, 1.5, 2.0, and 2.5). Dworak pathologic grading was used for treatment response assessment. Independent t-test was used to compare each texture feature between the treatment responder and non-responder groups. DFS was assessed with Kaplan-Meier method, and differences were compared with log-rank test. Cox proportional hazards models were constructed to predict prognosis based on stage, age, and each texture feature. Treatment responders (n = 32) showed significantly lower entropy, higher uniformity, and lower standard deviation in no filtration, fine (1.0), and medium (1.5) filter values. Entropy, uniformity, and standard deviation without filtration showed significant difference in DFS in Kaplan-Meier analysis (P = 0.015, 0.025, and 0.038). Homogeneous texture features (≤ 6.7 for entropy, > 0.0118 for uniformity, and ≤ 28.06 for standard deviation) were associated with higher DFS. Entropy, uniformity, and standard deviation were independent texture features in predicting DFS (P = 0.017, 0.03, and 0.036)

**Conclusions:**

Homogeneous texture features are associated with better neoadjuvant CRT response and higher DFS in patients with locally advanced rectal cancer.

## Introduction

The incidence of colorectal cancer is the fourth most common cancer in the United States and the third most common in South Korea [1, 2]. One-third of cases are rectal cancer. In cases of locally advanced rectal cancer, neoadjuvant concurrent chemoradiotherapy (CRT), followed by total mesorectal excision, has been widely performed as a standard treatment approach [3]. With preoperative CRT, pathologic complete response has been achieved in up to 16% of treated patients, and the local recurrence rate has been reduced to less than 10% [4–8]. Good treatment responders to neoadjuvant concurrent CRT show better recurrence-free survival, distant metastasis rate, and local recurrence rate than poor responders [9]; in patients who achieved clinical complete response after neoadjuvant CRT showed promising clinical outcomes with not only organ-preserving surgery including local excision but also with simple observation [10, 11]. Therefore, it is important to predict the CRT responses in patients with locally advanced rectal cancer for an optimal treatment plan.

Tumor heterogeneity, reflecting intratumoral variation in the cellularity, distribution of tumor vessels, extracellular matrix, hemorrhage, and necrosis, is a well-known feature of malignancy [12]. A potential non-invasive imaging biomarker, quantitative assessment of tumor heterogeneity by computed tomography (CT) texture analysis, has recently been investigated in colorectal cancer, lung cancer, head and neck squamous cell cancer, metastatic renal cell cancer, hepatic metastasis of colon cancer, and esophageal cancer [13–19]. Although several studies have predicted CRT responses with non-invasive imaging tools, such as high resolution magnetic resonance imaging (MRI) techniques [20], diffusion weighted images [21], and dynamic, contrast-enhanced MRI [22], to the best of our knowledge, no previous studies have investigated the relationship between patients’ outcomes after neoadjuvant CRT and the tumor heterogeneity assessed by texture analysis on pretreatment, contrast-enhanced CT. Thus, the purpose of our study was to evaluate the association of CT texture features of locally advanced rectal cancer with neoadjuvant concurrent CRT response and disease-free survival (DFS).

## Materials and methods

This single-center, retrospective study was performed in a tertiary general hospital. The institutional review board of Seoul National University Bundang Hopsital approved this study, and the requirement of informed consent was waived, IRB No.: B-1501-282-112.

### Clinical staging and patient inclusion

Digital rectal examinations, abdominopelvic CT, and pelvic MRI, with or without endorectal ultrasonography and chest CT, were performed for the clinical staging. The clinical stage grouping was derived from the TNM stage, according to the American Joint Committee on Cancer [23]. The locally advanced rectal cancer was defined as initial clinical stage of T3/4 or N1/2, without evidence of distant metastasis. Patients with locally advanced rectal cancer located within 10cm of the anal verge were eligible for inclusion in the neoadjuvant CRT.

**Fig 1:**
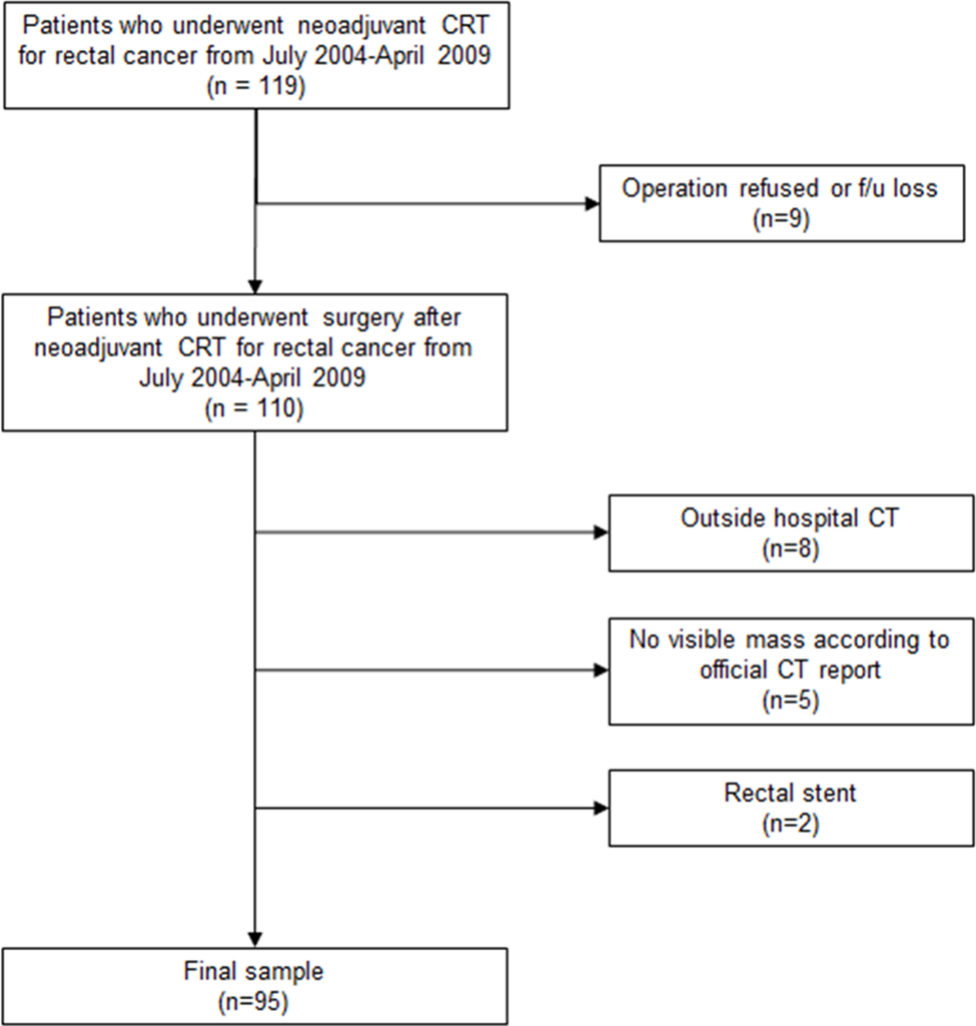
Patient flow diagram.

Patients who had locally advanced rectal cancer and completed neoadjuvant CRT from July 2004 through April 2009 were identified from a search of the hospital electronic database of medical records. A total of 119 consecutive patients with locally advanced rectal cancer who completed CRT were initially included in our study. Exclusion criteria for the final sample of this study were as follows: first, patients who refused surgery in our hospital were excluded. Second, patients who underwent initial CT outside the hospital were excluded, since the information and the quality of the CT protocol were unclear. Third, patients with a rectal stent or any prosthesis that could affect the measurement were excluded. Fourth, patients with unidentifiable rectal tumors on initial CT images were excluded. Of these 119 patients, nine patients who refused surgery, eight who underwent CT examination at another hospital, two who had a rectal stent, and five whose tumors were not visible on the initial CT images were excluded. A total of 95 patients were included in the final sample of the study (Fig 1).

### CT image acquisition

CT examinations were performed using 16- (n = 80) and 64- (n = 15) detector-row machines (Mx 8000 or Brilliance 64; Philips Medical Systems, Cleveland, OH). Intravenous nonionic contrast material (2mL/kg; iopromide, Ultravist 370: Bayer, Berlin, Germany) was administered via the antecubital vein, using a power injector (Stellant D, Medrad, Indianola, PA), at a rate of 3mL/sec. Bolus tracking software was used to trigger scanning, 60 seconds after the aortic enhancement reached a 150 Hounsfield unit threshold, to obtain portal venous phase axial images. CT scan data were acquired using the following parameters: 120kVp; 16 X 1.5 or 64 X 0.625mm collimation; a rotation speed of 0.5 seconds; and a pitch of 1.1 or 0.89. Tube current was automatically modulated (Dose-Right; Philips Medical Systems). All images were reconstructed by using filtered back projection and a body filter with a soft kernel algorithm was used. Axial and coronal images were reconstructed with 4mm thick thickness at 3mm intervals.

### Texture analysis

**Fig 2:**
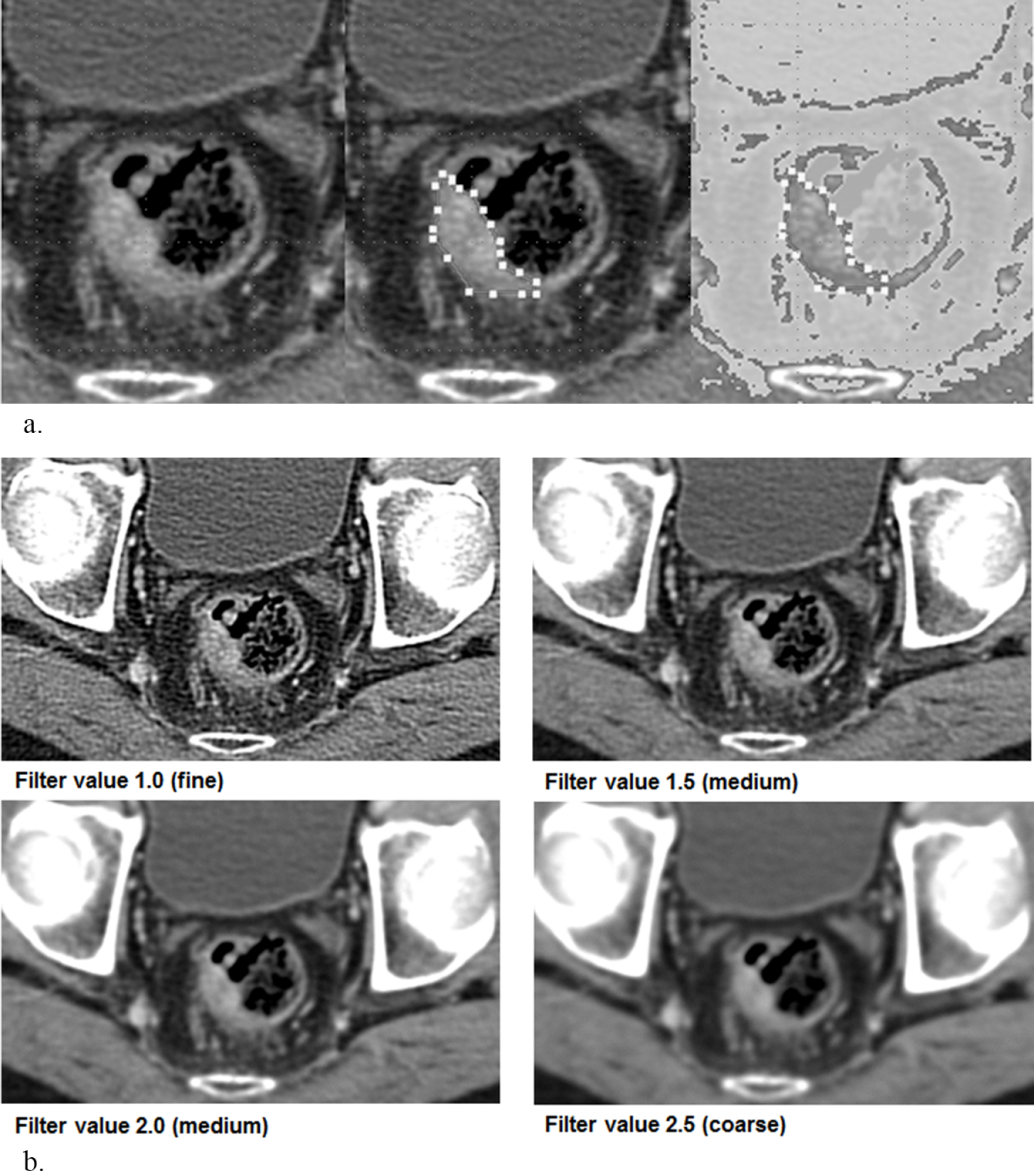
Texture analysis. (a) Manually outlining and filtering out the pixels with attenuation under -50 HU in locally advanced rectal cancer in 76-year-old man. (b) Corresponding images in the same patient applying LoG filters with fine, medium, and coarse filter values.

The pretreatment, contrast-enhanced CT study of each patient was retrieved from the institutional archive system and loaded to our independently developed software for texture analysis. All clinical outcomes were blinded during measurement. To draw the region of interest (ROI), an axial image representing the largest tumor area was selected, and the tumor margin was outlined manually (Fig 2) by the consensus of two radiologists (Y.H.K., and C.G.C., with 20 and four years of experience, respectively). If there was any ambiguity in outlining the tumor margin, the MRI image was reviewed. Intraluminal air regions were further excluded from the analysis by filtering out the pixels with attenuation under -50 HU.

Entropy, uniformity, kurtosis, skewness, and standard deviation of the pixel distribution histogram were calculated without filtration and with Laplacian of Gaussian (LoG) spatial filter with various filter values for fine (1.0), medium (1.5 and 2.0), and coarse (2.5) textures (Fig 2). LoG spatial filter was used to highlight features at different anatomic spatial scales, ranging from fine to coarse texture. A low filter value highlights the difference of fine anatomic details, while a high filter value allows coarse features to be highlighted. The methodology of applying the LoG filter with the aforementioned filter values refers to the previous reports of colon cancer texture analysis [13].

### Pathologic grading for CRT response

Histopathological evaluation of the surgical specimen was performed by one gastrointestinal pathologist (H.S.L., with 16 years of experience). After surgery, the specimens were fixed in formalin for 24 hours after inking of the circumferential resection plane. The whole tumor and mesorectum were serially sliced, axially, at 3mm intervals, and treatment response was assessed according to 5-point scale described by Dworak et al. [24]. The grading of regression was established as follows: Grade 0—no regression; Grade 1—dominant tumor mass with obvious fibrosis and/or vasculopathy; Grade 2—dominantly fibrotic changes with few tumor cells or groups (i.e., easy to find); Grade 3—very few tumor cells in the fibrotic tissue, with or without mucous substance (i.e., difficult to find); Grade 4—fibrotic mass only, without tumor cells (i.e., total regression or response).

### Patient follow-up

All patients underwent clinical follow-up according to our institutional protocol. The diagnosis of recurrence was made on the basis of an imaging study and, if possible, histopathologic examination was obtained by either biopsy or surgical excision. Disease-free survival (DFS) was defined as the time interval from the date of surgery to the first event of either recurrence or death from any cause. The outcome data in patients who were noted to be alive and free of recurrence at the time of their last follow-up were censored in the analysis. All of these data were retrospectively reviewed by one radiologist (C.G.C.).

### Statistical analysis

Statistical analyses were performed using the R software package (version 2.14.2; R Foundation for Statistical Computing, Vienna, Austria) and Stata 14.0 (StataCorp, College Station, TX). Patients were divided into either the responder (Grade 3 or Grade 4) or non-responder (Grade 0, 1, or Grade 2) group, according to Dworak’s pathologic grading. To illustrate whether the five texture features were related to the responder and non-responder groups, the mean values of the five texture features were compared by independent t-test between the groups. For a total of 25 combinations of five texture features and five filters, the optimal cutoff values that maximized the sum of sensitivity and specificity were used as a threshold for each texture value. To determine the association between the texture features and DFS, a log-rank test was performed, and a multivariable Cox proportional hazards model was used to adjust clinical confounders, such as CT stage and patient’s age. A P-value of 0.05 was considered significant.

## Results

### Patients demographics

95 patients (59 men; mean age, 61.1 years; age range, 36–85 years; 36 women; mean age, 60.2 years; age range, 35–78 years) with local advanced rectal cancer were included in the study (Table 1). Based on the CT findings, there were two patients with clinical stage I, 14 patients with stage II, and 79 patients with stage III cancers. Two patients with stage I, based on CT findings, were included for neoadjuvant CRT, because these patients were regarded as having locally advanced cancer, according to the MR imaging findings. Patients with stage IV cancer were not included. All patients underwent six weeks of neoadjuvant CRT, followed by surgery.

**Table 1:** Patient characteristics (n = 95).

Characteristic		Data
Age (y)—mean ± standard deviation		60.8 ± 11.6
	Male	61.1 ± 11.9
	Female	60.2 ± 11.2
Male		59 (62.1%)
Clinical staging according to CT findings		
	I^a^	2 (2.1%)
	II	14 (14.7%)
	III	79 (83.2%)
	IV	0 (0.0%)
CT machine		
	16 channel	15 (15.8%)
	64 channel	80 (84.2%)
CCRT regimen		
	XELOX	45 (47.4%)
	FOLFOX	46 (48.4%)
	Cetuximab/Irino/Xeloda	4 (4.2%)
Pathologic response		
	Grade 1	17 (17.9%)
	Grade 2	46 (48.4%)
	Grade 3	18 (19.0%)
	Grade 4	14 (14.7%)
Tumor recurrence		18 (18.9%)

^a^ Two patients were considered to have locally advanced cancer, according to the MRI findings.

Patients’ CRT responses included 17 patients with Grade 1, 46 with Grade 2, 18 with Grade 3, and 14 with Grade 4 according to Dworak’s pathologic grading. Eighteen of the 95 patients (18.9%) showed tumor recurrence during follow-up. The medial follow-up duration in all patients was 59 months (interquartile range, 41–78 months). The median DFS in all patients was 54 months (interquartile range, 28–75 months).

### Texture features

The texture analysis results of the locally advanced rectal cancer without filtration and with different filter values are summarized in Table 2. The treatment responder group (n = 32) showed significantly lower entropy, higher uniformity, and lower standard deviation (homogeneous features) in no filtration and fine (1.0) and medium (1.5) filter values than the non-responder group (n = 63). Skewness showed significant difference between the two groups in no filtration, medium (2.0), and coarse (2.5) filter values, and kurtosis did only in medium (2.0) filter value. The difference between the CRT responder and non-responder groups for the texture analysis measurement is summarized in Table 3.

**Table 2:** Results of texture feature analysis.

Filter Values	Entropy	Uniformity	Kurtosis	Skewness	Standard deviation
No filtration	6.76 ± 0.35	0.0113 ± 0.0027	0.87 ±1.73	-0.24 ± 0.62	30.52 ± 9.51
1.0 (fine)	7.26 ± 0.24	0.0079 ± 0.0015	1.66 ± 0.55	0.55 ± 0.62	44.64 ± 9.15
1.5 (medium)	6.85 ± 0.26	0.0107 ± 0.0020	0.95 ± 0.84	0.04 ± 0.50	32.18 ± 6.48
2.0 (medium)	6.58 ± 0.28	0.0129 ± 0.0026	0.89 ± 1.02	-0.52 ± 0.41	26.70 ± 5.41
2.5 (coarse)	6.45 ± 0.28	0.0142 ± 0.0031	1.05 ± 1.24	-0.81 ± 0.38	25.05 ± 4.96

Note: Data are mean ± standard deviation.

**Table 3:** Texture features of non-responder versus responder group after CRT without filtration and for various filter scale values depicting fine, medium, and coarse textures.

		Non-responder	Responder	P value
**No filtration**				
†	Entropy	6.84 ± 0.37	6.59 ± 0.22	< 0.001
†	Uniformity	0.0107 ± 0.0027	0.0125 ± 0.0021	< 0.001
	Kurtosis	0.96 ± 2.03	0.71 ± 0.86	0.513
†	Skewness	-0.08 ± 0.69	-0.55 ± 0.29	< 0.001
†	Standard deviation	32.80 ± 10.70	26.02 ± 3.71	< 0.001
**Filter 1.0 (fine)**				
†	Entropy	7.30 ± 0.20	7.18 ± 0.32	0.032
†	Uniformity	0.0076 ± 0.0012	0.0084 ± 0.0018	0.026
	Kurtosis	1.65 ± 2.17	1.66 ± 1.82	0.973
	Skewness	0.61 ± 0.58	0.44 ± 0.67	0.206
†	Standard deviation	46.02 ± 8.75	41.91 ± 9.45	0.038
**Filter 1.5 (medium)**				
†	Entropy	6.89 ± 0.25	6.77 ± 0.25	0.032
†	Uniformity	0.0104 ± 0.0019	0.0113 ± 0.0019	0.034
	Kurtosis	0.90 ± 0.82	1.05 ± 0.88	0.395
	Skewness	0.10 ± 0.47	-0.08 ± 0.55	0.094
†	Standard deviation	33.12 ± 6.54	30.34 ± 6.04	0.048
**Filter 2.0 (medium)**				
	Entropy	6.62 ± 0.28	6.50 ± 0.27	0.052
	Uniformity	0.0125 ± 0.0026	0.0136 ± 0.0026	0.07
†	Kurtosis	0.74 ± 0.95	1.18 ± 1.12	0.047
†	Skewness	-0.45 ± 0.40	-0.66 ± 0.40	0.017
	Standard deviation	27.36 ± 5.51	25.42 ± 5.06	0.1
**Filter 2.5 (coarse)**				
	Entropy	6.48 ± 0.28	6.38 ± 0.29	0.111
	Uniformity	0.0138 ± 0.0030	0.0148 ± 0.0032	0.13
	Kurtosis	0.88 ± 1.18	1.38 ± 1.31	0.061
†	Skewness	-0.75 ± 0.40	-0.92 ± 0.33	0.042
	Standard deviation	25.52 ± 5.00	24.12 ± 4.80	0.194

† P value < 0.05

Note: Data are mean ± standard deviation.

**Fig 3:**
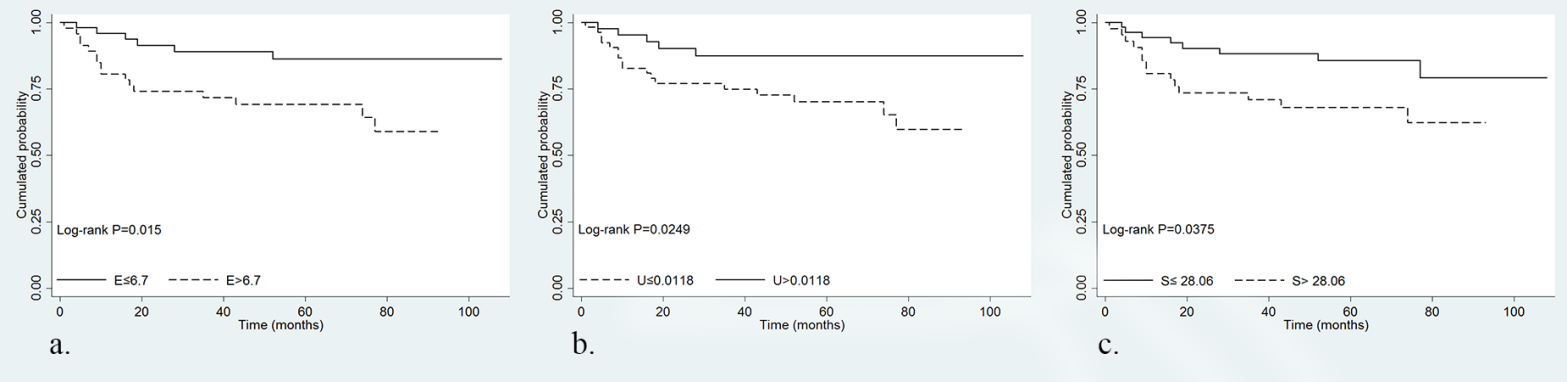
Kaplan–Meier curves according to texture features. Kaplan-Meier curves without filtration showed a significant difference in DFS for (a) entropy, (b) uniformity, and (c) standard deviation.

Log-rank tests for DFS showed significant difference for dichotomized entropy, uniformity, and standard deviation with no filtration (P = 0.015, P = 0.025, and P = 0.038). Homogeneous texture features (threshold values; ≤ 6.70 for entropy, > 0.0118 for uniformity, and ≤ 28.06 for standard deviation) were associated with higher DFS (Fig 3).

Collinearity was observed among the texture features. Strong negative correlation between entropy and uniformity (r = -0.98, P < 0.001) and strong positive correlation between entropy and standard deviation (r = 0.93, P < 0.001) were noted (Table 4). Due to the collinearity among entropy, uniformity, and standard deviation, each dichotomized texture feature was analyzed with clinical stage on CT and age, respectively, using the multivariable Cox proportional hazards model. Entropy, uniformity, and standard deviation were independently significant factors from the CT stage and age in predicting DFS (P = 0.017, 0.03, and 0.036) (Table 5).

**Table 4:** Spearman rank correlation for texture features without filtration.

	Entropy	Uniformity	Kurtosis	Skewness	Standard Deviation
Entropy	…	-0.98 (<0.001)	0.21 (0.044)	0.70 (<0.001)	0.93 (<0.001)
Uniformity	-0.98 (<0.001)	…	-0.12 (0.267)	-0.66 (<0.001)	-0.86 (<0.001)
Kurtosis	0.21 (0.044)	-0.12 (0.267)	…	0.51 (<0.001)	0.35 (0.001)
Skewness	0.70 (<0.001)	-0.66 (<0.001)	0.51 (<0.001)	…	0.76 (<0.001)
Standard deviation	0.93 (<0.001)	-0.86 (<0.001)	0.35 (0.001)	0.76 (<0.001)	…

Note: Data in parentheses are P values.

**Table 5:** Multivariable Cox proportional hazards regression analysis of texture features with CT stage and age as dependent covariate.

**Without filtration**	**Hazard ratio**	**95% confidence interval**	**P value**
Entropy	3.15	1.23, 8.07	0.017
Age	1.04	1.00, 1.09	0.037
CT stage^a^	3.70	0.84, 16.3	0.083
**Without filtration**	**Hazard ratio**	**95% confidence interval**	**P value**
Uniformity	0.33	0.12, 0.90	0.03
Age	1.05	1.00, 1.09	0.033
CT stage^a^	3.44	0.78, 15.13	0.102
**Without filtration**	**Hazard ratio**	**95% confidence interval**	**P value**
Standard deviation	2.54	1.06, 6.09	0.036
Age	1.04	1.00, 1.09	0.036
CT stage^a^	3.66	0.83, 16.14	0.086

^a^ Two patients with clinical stage I were merged to stage II, due to its small patient size.

## Discussion

The results of our study demonstrate the association of tumor texture features of pretreatment enhanced CT with neoadjuvant CRT response and the role of texture features as independent factors for predicting DFS in locally advanced rectal cancer. The treatment responder group showed homogeneous texture features, compared to the non-responder group, and the patient group with homogeneous texture features demonstrated higher DFS. Our results imply the potential of CT texture features as a non-invasive imaging biomarker predicting treatment response and patient prognosis.

In previous reports of CT texture analysis, heterogeneous texture features showing high entropy and low uniformity were associated with poorer overall survival in esophageal cancer, non-small cell lung cancer, and squamous cell carcinoma of the head and neck [14–16, 25]. Heterogeneity is a well-known character of malignant tumors, which is usually associated with aggressive tumor biology. The intratumoral heterogeneity induced by heterogeneous blood supply is associated with the presence of hypoxic areas, which can result in oxidative stress, promotion of survival factors, genomic instability, and treatment resistance [26–28]. In addition, a heterogeneous blood supply could be associated with a poor delivery of chemotherapeutic agent to low vascular areas, leading to treatment impairment [14]. Due to its low vascularity, on CT, the hypoxic or necrotic portion would show lower attenuation after contrast enhancement, compared with other portions of the tumor, and could be mainly attributable to the tumor heterogeneity in texture analysis.

Based upon our hypothesis, the result of our study that heterogeneous tumor texture was associated with poor DFS corroborates the fact that hypoxic cells are more resistant to ionizing radiation than those with normal levels in radiotherapy [29]. Such evidence has been reported in other cancers. Increased aggressive biologic behavior by heterogeneity, in terms of oxygen delivery, has been reported in prostate cancer [30], and resistance to radiotherapy in cervical cancer and head and neck cancer has also been reported [26, 27, 31].

We applied LoG spatial filter, with fine to coarse filter values, prior to texture analysis. It has been known through computer simulation that tumor vascularity is mainly attributable to the texture analysis in higher slice thickness on CT scan of three-dimensional vascular tree modeling [32]. Such results support the fact that, with a coarse scale LoG spatial filter, which accentuates the difference of coarse anatomic detail in the axial domain, tumor vascular supply plays a more important role in tumor heterogeneity [13]. However, unlike modeling, the true effect and interpretation of the application of LoG spatial filter on texture analysis is unclear in contrast-enhanced CT study. In our study, the information of the original CT tumor texture seemed to be lost as the filter was applied. DFS was statistically different only when the filter was not applied and, as the filter was applied in coarser scale, the statistical P-value of the difference between the responder and non-responder groups showed increasing tendency.

Our results appear to conflict with a previous report on colorectal cancer, in which homogeneous tumor texture was associated with poorer overall survival [13]. However, there were key differences between our study and the previous study. Our inclusion criteria limited the patient group to locally aggressive rectal cancer, while the previous study included primary colon and rectal cancer of various stages. Unlike rectal cancer, colon cancer does not necessitate neoadjuvant CCRT. Furthermore, treatment strategy varies according to the stage of colorectal cancer. Our study consisted of a homogeneous population, in terms of treatment modality, in that all patients completed the neoadjuvant CRT followed by total mesorectal excision.

Our study had several limitations. First, there is subjectivity in the process of manually outlining the tumor boundary. Further study may be needed to validate the reproducibility of our study results.

Second, some parameters were not interpretable. Skewness was statistically different in no filtration, medium filter value (2.0), and coarse filter value (2.5), while kurtosis showed difference only in medium filter value (2.0). Since skewness and kurtosis have no biological correlate, the biological validation and interpretation are unclear and interpretation must be done with caution. In addition, DFS was not associated with skewness and kurtosis.

Third, our measurement for heterogeneity on CT was done at the single largest cross-sectional area, rather than a whole-tumor analysis. Previous studies included whole-tumor texture analysis that is more representative of tumor heterogeneity than single largest cross-sectional area analysis in primary colorectal cancer [13]. However, we designed our study to analyze the largest cross-sectional area because, for some patients, outlining the tumor margin is ambiguous, even in the largest cross-sectional area and with the help of MRI. Furthermore, there were previous report that the measured entropy representing tumor heterogeneity was similar between both methods of measurement [33].

Fourth, the effects of the CT protocol on the tumor texture analysis still remain unclear. The tube voltage, current, reconstruction method, and contrast enhancement protocol may vary among institutions. Although the texture parameters are less sensitive to the CT acquisition factors [19], the effect of the reconstruction method and the acquisition time of the portal venous phase may need further validation. Therefore, the cutoff values that we measured may not be directly comparable with other institutions.

In conclusion, we observed that homogeneous texture features were associated with better neoadjuvant CRT response and higher DFS in patients with locally advanced rectal cancer. Our experimental study results advocate tumor texture analysis as a potential imaging biomarker for treatment response and prognosis and might offer additional information to clinicians for establishing treatment strategy.

## Supporting information

S1 Table Details of measured texture features. (XLSX)
